# Determining an Appropriate Time to Start Prophylactic Treatment with Intranasal Corticosteroids in Japanese Cedar Pollinosis

**DOI:** 10.3390/medsci7010011

**Published:** 2019-01-15

**Authors:** Takenori Haruna, Shin Kariya, Takaya Higaki, Sei-ichiro Makihara, Kengo Kanai, Yasutoshi Komatsubara, Aiko Oka, Kazunori Nishizaki, Mitsuhiro Okano

**Affiliations:** 1 Department of Otolaryngology-Head & Neck Surgery, Okayama University Graduate School of Medicine, Dentistry and Pharmaceutical Sciences, Okayama 700-8558, Japan; tharuna327@gmail.com (T.H.); skariya@cc.okayama-u.ac.jp (S.K.); takaya.higaki@gmail.com (T.H.); aoka0201@outlook.jp (A.O.); nishizak@cc.okayama-u.ac.jp (K.N.); 2 Department Otorhinolaryngology, Himeji Red Cross Hospital, Himeji 670-8540, Japan; yasutoshi0417@gmail.com; 3 Department Otorhinolaryngology, Kagawa Rosai Hospital, Marugame 763-8502, Japan; seiichiromakiharajp@yahoo.co.jp; 4 Department Otorhinolaryngology, Kagawa Prefectural Central Hospital, Takamatsu 760-0017, Japan; kengoo4665@yahoo.co.jp; 5 Department Otorhinolaryngology, International University of Health and Welfare School of Medicine, 4-3 Kozunomori, Narita 286-8686, Japan

**Keywords:** allergic rhinitis, double-blinded randomized placebo-controlled trial, fluticasone furoate nasal spray, increment cost effective ratio, intranasal corticosteroids, Japanese cedar pollinosis, post-onset treatment, prophylactic treatment, total naso-ocular symptom score

## Abstract

Prophylactic treatment with intranasal corticosteroids is effective for pollen-induced seasonal allergic rhinitis. However, the appropriate time to start this treatment remains unclear. We performed a double-blinded, randomized, placebo-controlled trial. Starting on 1 February 2014, patients with Japanese cedar pollinosis received either fluticasone furoate nasal spray (FFNS) for 8 weeks (Group A: *n* = 24), placebo nasal spray for 2 weeks followed by FFNS for 6 weeks (Group B: *n* = 23), or placebo for 4 weeks followed by FFNS for 4 weeks (Group C: *n* = 23). The primary endpoint was comparison of the total naso-ocular symptom score (TSS). Secondary endpoints including the increment cost effective ratio (ICER) were also determined. Continuous pollen dispersion began on the 24th of February. Therefore, Group A and Group B received 3-weeks and 1-week of prophylactic treatment, respectively, whereas Group C received post-onset treatment. During the peak pollen-dispersal period, significant differences in TSS were seen between the groups, particularly between Group A and C. The ICER of Group B vs. Group C was lower than that of Group A vs. Group C. These results suggest that long-term prophylactic treatment with FFNS is clinically the most potent treatment, whereas short-term prophylactic treatment is cost effective for pollen-induced allergic rhinitis.

## 1. Introduction

Prophylactic treatment, an early interventional or initial treatment, is recommended for patients with pollinosis who annually experience substantial naso-ocular allergic symptoms during the pollen season [1]. This treatment includes anti-histamines, anti-leukotrines and intranasal corticosteroids (INS) [2,3]. Of these medications, INS are selected in order to suppress pre-onset minimal persistent inflammation induced by a low exposure to pollens before continuous dispersal [4,5]. It is well-known that this treatment delays the onset and reduces the symptom severity of pollinosis [3,6,7,8,9,10,11]. 

Previous studies have reported starting prophylactic treatment with INS at time points ranging from 4 weeks before the pollen season to the beginning of pollinosis symptoms [3,6,7,8,9,10,11]. For example, we demonstrated that prophylactic treatment with a mometasone furoate nasal spray starting at 3 weeks before continuous pollen dispersal significantly delayed the onset of symptoms and alleviated symptom severity compared not only with placebo but also with post-onset treatment of patients with Japanese cedar pollinosis (JCP), which is the most common pollen-induced allergic rhinitis (AR) in Japan [11]. Therefore, the appropriate time to start prophylactic treatment with INS remains to be determined. 

In order to clarify the appropriate time to start treatment, we performed a three-armed, double-blinded, randomized, placebo-controlled trial using a fluticasone furoate nasal spray (FFNS) for JCP. These results, including economic evaluation, provide a basis for the use of INS for prophylactic treatment in pollen-induced AR.

## 2. Materials and Methods

### 2.1. Patients

Inclusion and exclusion criteria for the JCP subjects enrolled in this study were identical to those in our previous reports [9,11]. In brief, patients who were (a) male or female between the age of 16 and 65 years old and who had: (b) at least a 2-year history of JCP; (c) sensitization to Japanese cedar pollen as assessed by a skin prick test; and (d) the ability to accurately fill in diary cards, were included. Patients were excluded from the study if they had: (a) concomitant sinonasal disease that could potentially affect the outcome of the trial (e.g., nasal polyps, rhinosinusitis, nasal septum deviation); (b) concomitant treatment for AR caused by allergens other than Japanese cedar pollen; (c) rhinitis medicamentosa and non-infectious, non-allergic rhinitis; (d) cedar pollen-specific immunotherapy; (e) sinonasal surgery including laser vaporization of inferior turbinates within the past year; (f) medication with anti-allergic drugs including H1RA, chromones, glucocorticoids and decongestants within 2 weeks of the study initiation; (g) hypersensitivity to FFNS; (h) systemic infection including mycosis; or (i) were pregnant and/or breastfeeding.

### 2.2. Study Design

The study was a single-center, double-blinded, randomized, placebo-controlled, three-armed parallel group trial that was performed from 2013 to 2014. The primary endpoint was comparison of the total naso-ocular symptom score (TSS: the sum of sneezing, rhinorrhea, nasal congestion, watery eyes and itchy eyes scores) that were obtained from diary cards filled out from January 27 to 28 March in 2014 [9]. The secondary endpoints were comparisons of the total nasal symptom scores (TNSS: the sum of three nasal symptom scores), the total ocular symptom scores (TOSS: the sum of two eye symptom scores), the score of each symptom, the medication score (MS), the symptom-medication score (SMS) and safety. The rescue medication score was derived as follows: 0, no rescue medication taken; 1, use of either a levocetirizine dihydrochloride tablet (GlaxoSmithKline (GSK), Tokyo, Japan) or olopatadine hydrochloride eye drops (Kyowa Hakko Kirin Co., Tokyo, Japan); and 2, use of a levocetirizine tablet and olopatadine eye drops.

Screening visits were performed in December 2013. Prior to the study initiation, we estimated the sample size that would be required based on the mean and standard deviation values in the groups reported in our previous studies using INS [9,11]. In order to yield an 80% study power for the mean and standard deviation values of the active and placebo groups (0.48 ± 0.87 and 1.24 ± 1.20, respectively), a total of 20 subjects were required for the primary endpoint of the symptom score (α = 0.05). Based on these calculations, we determined that a sample size of 23 subjects per each group would be necessary, assuming that there would be a 5% drop-out rate [9,11].

Allocation concealment was granted by the central registry and computer-generated block randomization. Starting on 1 February 2014, patients received either FFNS (55 μg per nostril once a day in the morning) for 8 weeks (Group A: *n* = 24), a placebo nasal spray for 2 weeks followed by FFNS for 6 weeks (Group B: *n* = 23), or placebo for 4 weeks followed by FFNS for 4 weeks (Group C: *n* = 23). The placebo spray that was provided by GSK (Tokyo, Japan) had a white-colored lid and trigger. The lid and trigger of the FFNS bottle, which were initially cyan-colored, were changed to white to ensure that both FFNP and the placebo spray had the same appearance. Patients were allowed to use a levocetirizine tablet and olopatadine eye drops as rescue medications on demand. The study was approved by the Institutional Review Board of Okayama University Graduate School of Medicine, Dentistry and Pharmaceutical Sciences (approval: m21001). Written informed consent was obtained from each patient.

### 2.3. Economic Evaluation

For economic evaluation of prophylactic treatment of INS for pollinosis, the increment cost effective ratio (ICER) to post-onset treatment (Group C) was compared between the long-term (Group A) and the short-term (Group B) prophylactic treatments [12]. Effectiveness was calculated as the sum of the TSS over the whole medication period (February to March). Costs were calculated by the sum of the drug expense for each subject (FFNS: 2040 yen/bottle; levocetirizine: 105 yen/tablet; olopatadine eye drops: 198 yen/bottle in 2014). Since a lower TSS shows better effectiveness, the ICER was determined as follows:

ICER = (Cost _group A or B_ − Cost _group C_)/(Effectiveness _group A or B_ − Effectiveness _group C_)

### 2.4. Measurement of Pollen Dispersion

From January 7, 2014, daily Japanese cedar and Japanese cypress pollen counts were performed using Durham samplers that were installed on the rooftops of the Okayama University Hospital buildings. The first day of the major pollen period was defined as the first of two consecutive days for which >1 grain/cm^2^ was counted [9].

### 2.5. Statistical Analysis

Values are expressed as the median for each subject group. A non-parametric Kruskal-Wallis test followed by a Dunn’s test was used to compare the data between groups. Statistical analyses were performed using GraphPad Prism (GraphPad software, Inc., La Jolla, CA, USA) version 9.2, with *p* < 0.0.5 considered to be significant.

## 3. Results

### 3.1. Patient Population

Out of the 72 patients that were screened, a total of 70 patients fulfilled the inclusion criteria. After enrollment in the study, patients were randomized into one of three groups, with 24 (Group A) or 23 (Group B and C) patients per group. Group A received FFNS for 8 weeks; Group B received placebo for 2 weeks flowed by FFNS for 6 weeks; and Group C received placebo for the first 4 weeks followed by FFNS for 4 weeks. Sixty-nine subjects (98.6%) completed the study. One patient in Group A dropped out due to de novo pregnancy. Therefore, the full analysis set consisted of 69 patients (23 patients in each group). The treatment groups were comparable with respect to demographic characteristics, which included age, sex, disease duration, and severity of Japanese cedar/cypress pollinosis (JCCP) (Table 1).

### 3.2. Fluctuation of Cedar Pollen Dispersion

**Figure 1:**
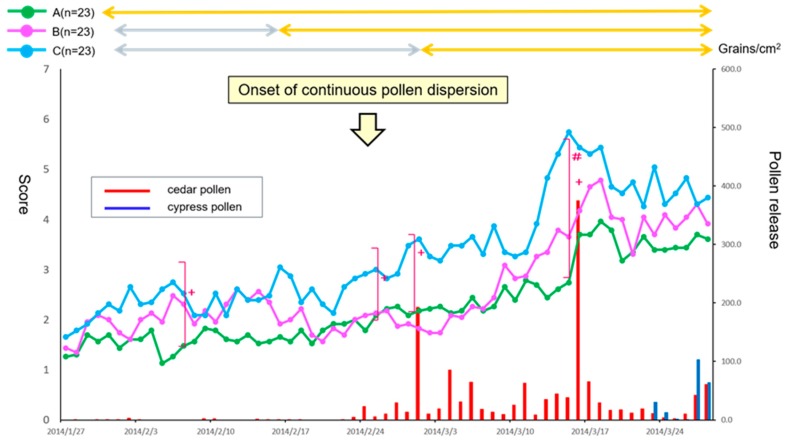
Comparison of primary endpoint efficacy between the treatment groups and fluctuation of cedar pollen dispersal. The daily reflective total naso-ocular symptom score (TSS) from baseline in patients with 3-weeks of prophylactic treatment (Group A: green), 1-week of prophylactic treatment (Group B: magenta) or post-onset treatment (Group C: cyan) with fluticasone furoate nasal spray (FFNS) is shown. Results are expressed as median values. The fluctuation in cedar pollen dispersal is shown by the red bars. A Kruskal-Wallis test was used to compare data between the three groups (+ *p* < 0.05). A Dunn’s test was further used to compare data between each group (# *p* < 0.05).

During 2014, the first dispersion (sporadic) of the Japanese cedar pollen occurred on January 17, with major continuous dispersion beginning on 24 February. The highest cedar dispersion was seen on 17 March, with 374.3 grains/cm^2^/day. The total cedar pollen count during the study period was 1419.3 grains/cm^2^ (Figure 1). The treatment drugs and placebos were prescribed only once for all patients at the same time (January 27–31).

### 3.3. Primary Endpoint Efficacy

Naso-ocular symptoms determined by the TSS differed significantly between the groups on 6 days over the medication period of 56 days (6, 24, 28 February, and 13–15 March: *p* < 0.05 by a Kruskal-Wallis test). In addition, a Dunn’s test indicated that Group A showed significant symptom alleviation on 3 days (13–15 March) as compared with Group C (*p* < 0.05). A clinically important difference (more than 1.5 points) was also seen between Group A and Group C (Figure 1) [13]. 

### 3.4. Secondary Endpoint Efficacy

**Figure 2:**
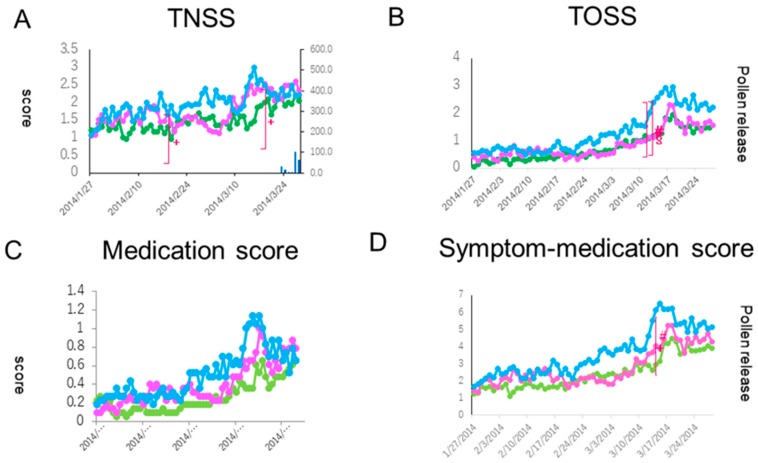
Comparison of secondary endpoint efficacies of the total nasal symptom score (TNSS, (**A**)), the total ocular symptom score (TOSS, (**B**)), the medication score (**C**), and the symptom-medication score (**D**) between the treatment groups. The lines, symbols and bars are explained in Figure 1. +: *p* < 0.05 (Kraskal-Walis test); #: *p* < 0.05 (A vs. C); §: *p* < 0.05 (B vs. C (Boferoni/Dunn test)).

A Kruskal-Wallis test showed a significant difference in the TNSS between the groups in two out of 56 days (19 February and 14 March: *p* < 0.05). Although a Dunn’s test showed no significant differences between the groups, a clinically important difference (more than 0.9 point) between Group A and B and between Group A and C was seen on the latter day in the peak pollen dispersal period (Figure 2A).

**Figure 3:**
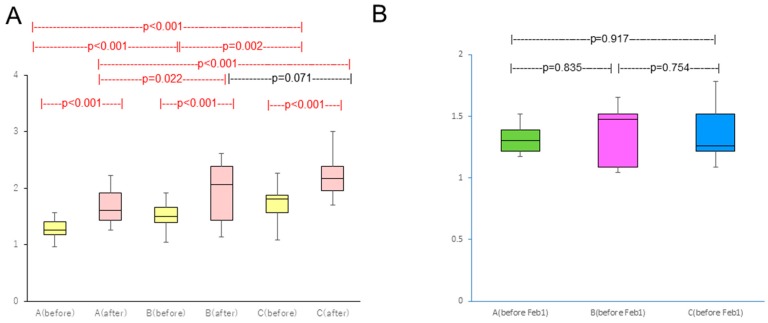
(**A**) Comparison of TNSS between before (27 January–23 February) and after pollen dispersion (24 February–28 March); (**B**) Comparison of TNSS between groups before study enrollment. Red bars indicate statistical significance.

There was significant difference between before and after pollen dispersion in all groups (*p* < 0.001) (before: 27 January–23 February, after: 24 February–28 March) (Figure 3A).

**Figure 4:**
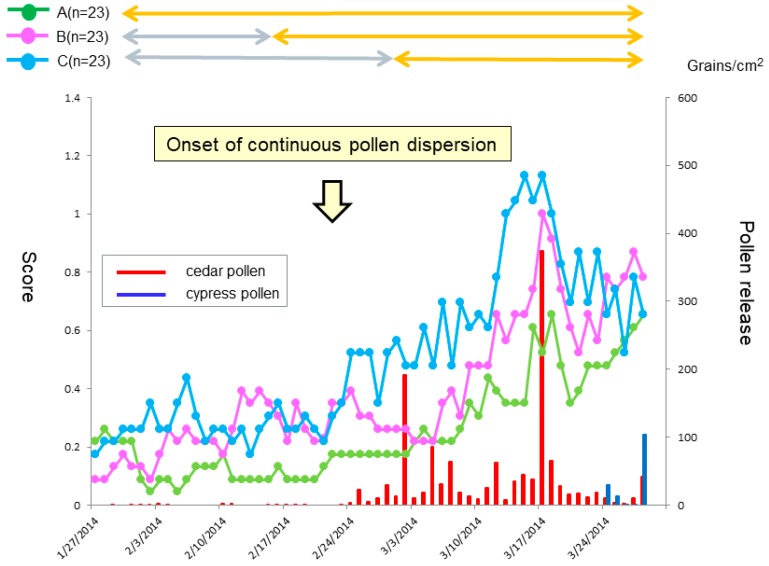
Daily scores reflective of quality of life (QOL) score from baseline. The lines, symbols and bars are explained in Figure 1.

Ocular symptoms used to determine the TOSS during the peak pollen dispersal periods (14 and 15 March) also showed a significant difference between the three groups (*p* < 0.05). A Dunn’s test further indicated a significant symptom alleviation in the prophylactic treatment groups (Group A and Group B) as compared with the post-onset treatment group (Group C) (both *p* < 0.05) (Figure 2B). In quality of life (QOL) score, no significant differences between the groups were shown (Figure 4).

Patients were allowed on-demand use of a levocetirizine tablet and olopatadine eye drops during the study period. The medication score (MS) showed a significant difference on 14 March between the groups (*p* < 0.05). A Dunn’s test further indicated a significant fewer drug consumption in the prophylactic treatment groups (Group A and Group B) as compared with the post-onset treatment group (Group C) (both *p* < 0.05) (Figure 2C). A similar result for Group A was seen in the SMS, where a significant difference was seen on 14 March between Group A and Group C (*p* < 0.05 by a Dunn’s test) (Figure 2D).

Group A took 2.8 levocetirizine dihydrochloride tablets (315 yen/tablet) and 1.00 olopatadine hydrochloride eye drops bottle (198 yen/bottle), Group B took 2.4 levocetirizine dihydrochloride tablets and 0.96 olopatadine hydrochloride eye drops bottle, and Group C took 4.5 levocetirizine dihydrochloride tablets and 1.43 olopatadine hydrochloride eye drops bottle (Table 2). No symptom severity difference existed between groups before study enrollment (Figure 3B).

### 3.5. Courses of Individual Symptoms

**Figure 5:**
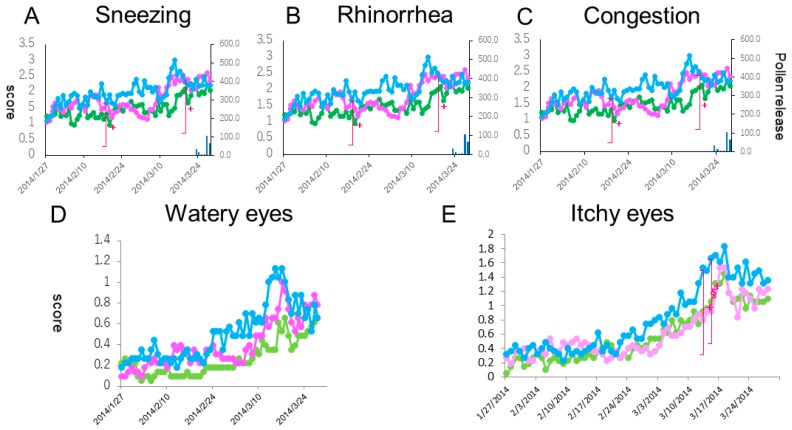
Daily scores reflective of sneezing (**A**), rhinorrhea (**B**), nasal congestion (**C**), watery eyes (**D**) and itchy eyes (**E**) from baseline. The lines, symbols and bars are explained in Figure 1. +: *p* < 0.05 (Kraskal-Walis test); §: *p* < 0.05 (B vs. C (Boferoni/Dunn test)).

When we compared the courses of five individual naso-ocular symptoms between the three groups, significant differences in the scores of sneezing, watery eyes and itchy eyes were seen on 6, 3 and 2 days, respectively, out of the 56 days (*p* < 0.05) (Figure 5A,D,E). No significant differences in rhinorrhea and nasal congestion scores were seen over the medication periods (Figure 5B,C). During the peak pollen period (mid-March), clinically important differences (more than 0.3 point) in sneezing and ocular symptoms were seen between the prophylactic treatment (Group A and B) and the post-onset treatment (Group C) groups. In sneezing, such a clinically important difference was seen between the 1-week prophylactic treatment group (Group B) and the 3-week prophylactic treatment group (Group A). 

### 3.6. Safety

All the treatments were well-tolerated and had a similar safety profile (Table 3). While there were eight, ten and 19 mild adverse events reported in Group A, B, and C, respectively, none of the events caused any of the patients to drop out of the study.

### 3.7. Increment Cost Effective Ratio

The sum score of the TNSS over the study period for Group A, B and C was 130, 150 and 191, respectively. The medication cost for Group A, B and C was 8673, 6633 and 5001 yen, respectively (Table 2). The ICER of Group A vs. Group C was calculated as follows: (8673 − 5001)/(130 − 191) = 60.2 (yen/score). The ICER of Group B vs. Group C was similarly calculated as follows: (6633 − 5001)/(150 − 191) = 39.8 (yen/score). The value of the latter ICER was lower than that of the former ICER.

## 4. Discussion

The present study demonstrated a significant difference in the control of pollinosis between the prophylactic and the post-onset treatments with FFNS. In particular, the 3-weeks of prophylactic treatment with FFNS showed a substantial effect on the control of nasal symptoms as compared with the post-onset treatment. On the other hand, the present study clarified for the first time that the ICER for the post-onset treatment was lower for the 1-week prophylactic treatment with FFNS than that for the 3-weeks of treatment. These results suggest that long-term prophylactic treatment with FFNS is clinically the most potent treatment, especially for nasal symptoms, whereas short-term prophylactic treatment is cost effective for Japanese cedar pollinosis.

Although prophylactic treatment with INS is effective and safe for pollinosis, little is known about the appropriate time to start this treatment [3,6,7,8,9,10,11]. Morelli et al. showed the efficacy and safety of 4-week prophylactic treatment with budesonide topical nasal powder in patients with grass pollinosis [3]. Graft et al. also showed the efficacy and safety of 4-week prophylactic treatment with a mometasone furoate aqueous nasal spray (MFNS) in patients with ragweed pollinosis [6]. Pitsios et al. showed the efficacy and safety of 2- to 4-weeks of prophylactic treatment with MFNS in patients with grass, Parietaria and/or olive pollinosis [7]. We have previously reported that 3-weeks of prophylactic treatment with MFNS is effective for JCP as compared with the placebo treatment [9,11]. On the other hand, Yamamoto et al. demonstrated that 1-week of prophylactic treatment with MFNS significantly alleviated late-phase symptoms as compared with treatment with fexofenadine after exposure to Japanese cedar pollen in environmental challenge chambers [10]. The present trial demonstrated for the first time that a significant difference in the TSS, which was set as a primary endpoint, was seen between the prophylactic and the post-onset treatments with FFNS, and that no significant or clinically important difference was seen between short- (1-week) and long-term (3-weeks) prophylactic treatments [13]. On the other hand, when we analyzed individual symptoms, a clinically important difference was seen in the sneezing score between 1-week and 3-weeks of prophylactic treatment. These results suggest that short-term prophylactic treatment with INS is sufficient for controlling JCP in general; however, longer prophylactic treatment may be considered for patients who have difficulty with nasal symptoms, especially sneezing, every season. 

It is suggested that INS has good effects on sneezing, and INS was effective for ocular itching through the naso-ocular reflex. Also, during the preseanoal priod (around 8 February), there was a significant difference in symptom scores among the group. It is suggested that in the beginning of February a small amount of pollen dispersion was found and the patients might have been affected by pollen stimulation.

One of the bases of prophylactic treatment with INS for pollinosis is the control of pre-onset minimal persistent inflammation that is present in the initial dispersion of pollen prior to the onset of symptoms, and which contributes to hyperreactivity and subsequently to the onset of full-scale symptoms [4,5]. We have recently reported that pre-onset activation of eosinophils and mast cells following repeated nasal provocation with Japanese cedar pollen extracts are suppressed by treatment with FFNS that is initiated one day before the first provocation [5]. Our current result is consistent with that report, and confirms a rapid and substantial potency of FFNS for prophylactic use in seasonal AR.

Intranasal corticosteroids reduce ocular symptoms such as watery and itchy eyes through several mechanisms including reduction of nasal-ocular reflexes [14,15]. We have previously reported that prophylactic treatment with MFNS significantly suppressed deterioration of ocular symptoms as compared with not only placebo treatment but also with post-onset treatment with MFNS in JCP [11]. The present result confirmed the efficacy of prophylactic treatment of INS for ocular symptoms and suggests again that 1-week prophylactic treatment with INS is long enough to control the ocular symptoms in JCP.

Economic evaluation of new medical interventions is of growing concern due to limited health care resources. Economic evaluation includes cost minimization analysis, cost-effective analysis, cost-utility analysis and cost-benefit analysis [16]. Of these analyses, cost-effective analysis by determination of the ICER is preferentially investigated in allergic rhinitis. For example, Verheggen et al. used a Markov model with a time horizon of 9-years and predicted costs and quality-adjusted life year (QALY) for 3-year sublingual immunotherapy (SLIT) and subcutaneous immunotherapy (SCIT) treatment of patients with grass pollinosis in Germany They estimated that the ICER of SLIT to SCIT was €12.593 per QALY, which suggested that SLIT with a 5-grass tablet was cost-effective relative to SCIT with allergoid compounds [17]. Goodman et al. investigated the cost-effectiveness of the second-generation antihistamines levocetirizine and desloratadine, and the antileukotriene montelukast, in relieving nasal symptoms using TNSS as the measure of effectiveness and prescription therapy and rhinitis-related physician office visit as the measure of costs. They demonstrated that the average cost-effectiveness ratio of levocetirizine was significantly lower than that of desloratadine or montelukast [12].

Since the present study was designed as a three-armed parallel group trial, the ICER of 1-week and 3-week prophylactic treatment with FFNP could be compared to post-onset treatment. Our results showed that the ICER of the 1-week prophylactic treatment to post-onset treatment was lower than that of the 3-week prophylactic treatment, which suggested that short-term prophylactic treatment with INS is a cost-effective therapy for controlling allergic symptoms in JCP. Without placebo treatment, the symptom score might have been worse, and therefore, cost effectiveness difference might have been more significant in the 3-week prophylactic treatment group compared to the post-onset treatment group. On the other hand, our study has a limitation in terms of cost estimation because the cost did not include indirect medical costs such as loss of productivity or intangible costs such as mental anguish [18]. Direct costs other than drug expenses, such as transportation expenses to our hospital, appeared to be similar in each group since all subjects visited the hospital three times during the study period and all subjects lived in Okayama city and its suburbs.

## 5. Conclusions

Long-term prophylactic treatment with FFNS is clinically the most potent treatment, whereas short-term prophylactic treatment is more cost effective for Japanese cedar pollinosis. These results may provide a basis for determination of the appropriate time to start prophylactic treatment in pollinosis.

## Figures and Tables

**Table 1:** Patient demographics. * One patient in Group A dropped out due to de novo pregnancy.

		Group A	Group B	Group C
Number		24 *	23	23
Age	Mean ± SD	36.3 ± 8.9	38.2 ± 8.6	43.1 ± 9.7
	Range	21–48	19–50	28–69
Sex (male/female)		7/17 *	5/18	5/18

SD: standard deviation

**Table 2:** Sum of the total naso-ocular symptom score and the drug cost over the study period for each treatment group.

	Sum of Naso-Ocular Symptom Scores from February to March	Drug Cost			Sum of Drug Cost
		FFNS (2040 yen/bottle)	Levocetirizine (105 yen/tablet)	Olopatadine Eye Drop (198 yen/bottle)	
Group A	130	4 bottles (8160 yen)	2.8 tablets (315 yen)	1.00 bottle (198 yen)	8673 yen
Group B	150	3 bottles (6120 yen)	2.4 tablets (315 yen)	0.96 bottle (198 yen)	6633 yen
Group C	191	2 bottles (4080 yen)	4.5 tablets (525 yen)	1.43 bottles (396 yen)	5001 yen

**Table 3:** Adverse events reported during the study regardless of their relationship to the treatment.

	Group A	Group B		Group C	
		Placebo	FFNS	Placebo	FFNS
Cold	7	3	3	3	1
Urticaria	1				
Headache		1		1	1
Cough		1		3	
Pharyngeal discomfort			1	3	
Itch of face			1		
Fever					1
Nasal bleeding				1	
Ear pain				2	
Aphthous ulcer				1	
Sore throat					1
Oral discomfort				1	
